# Evaluation of the Effect of Light and Scanning Time Delay on The Image Quality of Intra Oral Photostimulable Phosphor Plates

**DOI:** 10.2174/1874210601711010690

**Published:** 2017-12-29

**Authors:** Amir Eskandarloo, Arman Yousefi, Setareh Soheili, Karim Ghazikhanloo, Payam Amini, Haniyeh Mohammadpoor

**Affiliations:** 1 Department of Oral and Maxillofacial Radiology, Dental School, Hamadan University of Medical Sciences, Hamadan, Iran; 2 Department of Oral and Maxillofacial Radiology, Dental School, Ardabil University of Medical Sciences, Ardabil, Iran; 3 Department of Pediatric Dentistry, Dental School, Ardabil University of Medical Sciences, Ardabil, Iran; 4 Department of Medical Physics, Paramedical school, Hamadan University of Medical Sciences, Hamadan, Iran; 5 Department of Epidemiology and Reproductive Health, Reproductive Epidemiology Research Center, Royan Institute for Reproductive Biomedicine, ACECR, Tehran, Iran; 6 Private practice, Kelachai, Iran

**Keywords:** Contrast, Spatial resolution, Photostimulable phosphor plates, Digital image, Environmental light, Density

## Abstract

**Background:**

Nowadays, digital radiography is widely used in dental practice. One of the most common types is Photo Stimulated Phosphor Plate (PSP).

**Objective:**

The aims of this experimental study were to evaluate the impacts of different combinations of storage conditions and varying delays in reading of digital images captured using PSPs.

**Methods:**

Standardized images of a step wedges were obtained using PSPs from the Digora digital systems. Plates were exposed and immediately scanned to produce the baseline gold standard. The plates were re-exposed and stored in four different storage conditions: white light, yellow light, natural light environment and dark room, then scanned after 10 and 30 minutes and 4 and 8 hours. Objective analysis was conducted by density measurements and the data were analyzed statistically using GEE test. Subjective analysis was performed by two oral and maxillofacial radiologists and the results were analyzed using McNemar’s test.

**Results:**

The results from GEE analysis show that in the natural light environment, the densities in 10 minutes did not differ from the baseline. The mean densities decreased significantly during the time in all environments. The mean densities in step 2 for the dark room environment decreased with a slighter slope in comparison to yellow environment significantly.

**Conclusion:**

PSP images showed significant decrease in the density in plates scanned for 10 minutes or longer after exposure which may not be detected clinically. The yellow light environment had a different impact on the quality of PSP images. The spatial resolution did not change significantly with time.

## 1 INTRODUCTION

Nowadays, digital radiography is widely used in dental practice. Due to some special assets such as shorter exposure to radiation, capability to use pecular software to adjust the quality of images, simple way of storing and other spectacular advantages, conventional radiography has been gradually replaced by digital radiography. Several systems have been introduced for intraoral digital radiography. One of the most common types of these systems is Photo Stimulated Phosphor Plate (PSP). PSP image receptor systems are differentiated from other types by peculiar features of the plates: thin structure and flexibility and by the absence of a connecting wire. These features facilitate placement of the receptor into the mouth. Other advantages of PSP systems comprise being available in exactly the same size as conventional films having a wider dynamic range, which produces better quality radiographs compared with other digital sensors. The major drawbacks of PSPs are, the remaining energy is stored in the plate after scanning, lower quality owning to repeated applying, and handling and scanning of the plates are time consuming [1, 2]. Size and flexibility of Storage phosphor plates (SPPs), is similar to that of conventional films, and undoubtedly have been regarded as one of the most proper alternates for conventional films with great diagnostic accuracy and broader latitude [3].

PSP plates store absorbed x-ray energy in crystal structure in the form of trapped electron, and constitute a latent image. This deposited energy can be released if stimulated by additional light with proper wavelength. As the rate of difference between stimulating light and phosphorescent light wavelengths differs, the two might be differentiated, and the illumination can be determined as a measure of the quantity of x-ray energy that has been deposited in the material [4, 5]. These plates consist of polyester base coated with europium-activated barium fluorohalide crystals. By trapping electrons produced by an exposure to sufficiently energetic source of radiation, the phosphor crystal lattice is capable of storing a notable magnitude of the X-ray energy, hence producing a latent radiographic image. Stimulating red light is next applied to move the electrons into metastable states from which energy transfer will create a visible light which is accumulated and digitized [6, 7].

After exposure, plates should be processed quickly because the electrons which are in metastable state inevitably move to the ground state over the time. The rate of releasing the electrons is tremendously short after exposure. The rate differs based on the composition of the phosphor layer and the storage condition such as temperature and light. Some crystals release 23% of their trapped electrons after 30 minutes and 30% after 1 hour [5-8]. In clinical practice, it is not always possible to scan a PSP plate straight away after exposure, in addition, the conditions of storage are variable. So the objective of the current study, therefore, was to evaluate the effects of storage conditions (different environmental light) and scanning time delay on the quality of the PSP images.

## 2 MATERIALS AND METHODS

**Fig. (3A and B):**
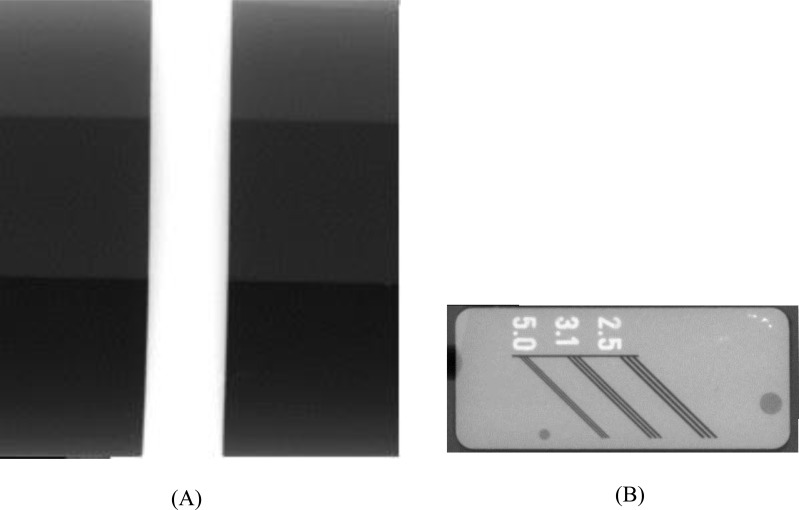
Images of contrast (A) and spatial resolution (B) phantoms.

**Fig. (4):**
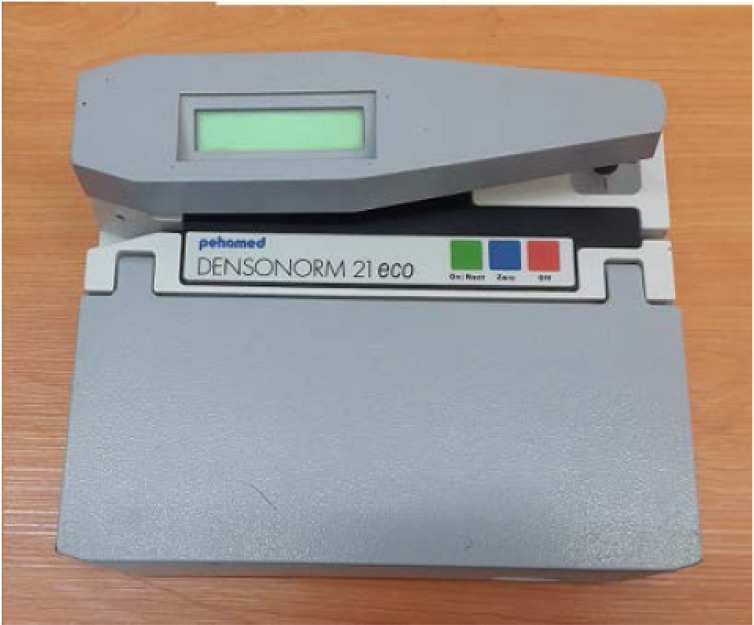
Densitometer which determines density.

In an *in vitro* study, the standard images from two phantoms (Fig . **1**). (Pehamed, Rosenberg, Germany) by PSP receptors (Fig . **2**) (Soredex, Tunsula, Finland), were prepared. One of the phantoms which contains a step-wedge, assessed the contrast and pixel density and had 3 steps; two of them were composed of polytetrafluoroethylene and the other was made up of copper. The other phantom considered spatial resolution that had 3 types of line pairs (2.5 / 3.1 / 5.0) in terms of the number of line pairs per millimeter (Fig. **3**). Each PSP image was captured on a new plate, with size2 (30mm× 40mm). Before each exposure, the plates were erased to eliminate residual images by means of the strong light source. The exposure setting was determined to see all the step-wedges and lines on the radiograph. The plates were exposed for 0. 6 seconds at 60 kVp, 10 mA, with focus-to-receptor distance 20 cm, using an X-ray unit (Planmeca,Tunsula, Finland). After scanning of PSPs, by Digora optim scanner (Soredex, Tunsula, Finland), Scanora imaging software was applied to view the resultant radiographic images, which were then stored in TIFF format and later, radiographs were printed by a printer (Kodak 58-50, New York, USA). To create the gold standard images, PSP plates were exposed and then were immediately scanned. Plates were again exposed and placed in 4 different environments (white light, yellow light, natural light and dark room), for 10 and 30 minutes and 4 and 8 hours and then scanned. PSPs in this period were within its coverage. Ambient light intensity was adjusted by using a Digital Lux Meter (model ST-1200) to the range of 150 Lux. The light intensity was adjusted according to the average of light intensity in 5 radiology clinics that was calculated before. The resulting images of phantom contrast (c) were assessed to consider the grey level changes in all steps by a densitometer (Pehamed, Densonorm, Rosenberg, Germany) (Fig. **4**)). The resulting images of the phantom spatial resolution(s) were evaluated by two oral and maxillofacial radiologists) by visual inspection. So that they assessed that how many couples of 3 listed pair lines were visible in each image separately. *SPSS* statistical analysis software (ver.21 *SPSS* inc. Chicago, IL) was applied to determine the signal fading of areas on the same steps, to assess the grey value differences due to delay in scanning time and effect of different environments. In addition, the number of visible pair lines in each image was determined. At last, the data was analyzed by GEE (Generalized Estimating Equation) model and McNemar's test.

### 2.1 Statistical Analysis

The mean and standard deviation were used to describe the continuous variables. Frequency and percentage were reported for the categorical variables. The densities were recorded longitudinally over time and hence, longitudinal methods were used to analyze the data. A population average method, Generalized Estimating Equation model (GEE) was applied to take into account the natural variance of repeated measurements over time. The most important feature of GEE models is the population average interpretation of the results so that an average trend of response variable over the time can be determined using multiple subject trends [9]. Using this method, one can evaluate the effect of different factors and covariates on the response variable. The reference category at the baseline is assumed as the baseline measurements. The reference category for the trend is assumed as the dark room environment. The estimated coefficients are the estimated mean difference of categories with the baseline measurements using the GEE method. According to the sufficient sample size, unstructured covariance pattern was used to take into account the association between the repeated measurements. The interpretation of the coefficients is population average. The coefficients resulted from the GEE approach can be interpreted both within and between the subject effects. The interaction term provides the difference between the groups over the time. The time was assumed in minutes.

## 3 RESULTS

The mean and the standard deviation of densities at the baseline as well as 10, 30, 240 and 480 minutes are shown in Table **1**. This table shows the results from GEE analysis where the mean densities between the baseline (immediately scanned plates) and the other time points are compared. The comparisons are presented in three types of error levels. The results in Table **1** show that in the natural light environment, the densities in 10 minutes did not differ from the baseline in all the steps. Moreover, the mean densities at 10 minutes in the yellow and white light environments were not statistically different from the baseline in step 2. Other comparisons with the baseline values were observed to be statistically significant.

The results of GEE analysis method are presented in Table **2** showing the trend of mean densities during 8 hours. As observed, the mean densities decreased significantly during the time in all environments for the three steps. No significant difference in the trend was found between the environments in step 1and 3. The densities during the time are presented in Figs. (**5**-**7**).

The mean densities in step 2 for the dark room environment (slope=-0.040, *p*<0.001) decreased with a slighter slope in comparison to yellow environment (slope difference with dark room Env=-0. 036, *p*=0.018), significantly. Hence, the decreasing slope for yellow environment was 0.076. The decrease in slopes for the other two environments was the same as for dark room environment statistically.

Based on Table **3**, the estimated mean density in step 1 and at the baseline is 1.988 while it was significantly higher than step 2(mean difference=0.237 and *p*<0.001) and step 3(mean difference=0.7 and *p*<0.001). In contrast to the baseline, the densities were higher in step 2 (mean difference over the time=0.0002, *p*<0.001) in comparison to section 1 over the time.

The spatial resolutions (observed line pairs) shown in Fig. (**8**), Using McNemar’s test, show that however the spatial resolution after 30 minutes decreased, it was not significant.

## 4 DISCUSSION

Digital radiography was introduced in 1981 by Fuji with the first commercial computed radiography (CR) imaging system, and there has been continuous improvement in this new technology. A wafer-thin phosphor crystal layer of the BaFX:Eu2 compound, resulted better image formation by PSP plates with less scattering, increased energy absorption and improved spatial resolution. Like conventional films, the PSP plates are stimulated by ionizing radiation, but require scanning process by special hardware and also peculiar software. Images are stored in a computer system to facilitate access required every time [10, 11]. In clinical practice, it is not always possible to scan a PSP plate straight away, and therefore, a delay between exposure and scanning process would be inevitable. This situation usually occurs in full-mouth series, because there usually is a gap time between the first and the last captured images. Furthermore, this time lag can happen when there is network problem or power outage. The states in which the phosphor plates are stored are also presumably different. This study was therefore carried to understand if delayed scan and variable storage conditions of phosphor plates bring about deprivation of image quality. Several studies related to signal fading have been conducted [1-3, 7, 8, 10-16]. They concluded that the density decreased with scan delay. In this study, our quantitative analysis of pixel intensity value level (pixel density) in all 3 steps did demonstrate a decline in density with delay in scanning time. Despite, there was a statistically significant change in the average pixel density, this did not seem to prevail the spatial resolution of the images of PSP plates significantly at the clinical level. Corresponding findings were reported by Dan *et al.* [9], who also noted signal differences in PSP images over time; nevertheless, these changes did not clinically affect the spatial resolution of images. However, they did not store plates in different environments. According to our results, keeping the plates in natural light environment did not affect the density until 10 minutes and after 10 minutes, density was reduced significantly. This trend was also found in some steps of step-wedges in other environments, which reflects the importance of time in scanning procedure. Although this finding is in accordance with a previous reports by Aktan *et al.* [2], Sogur *et al*. [3], and Akdeniz *et al*. [11], it is different to the findings of Martins *et al*. who demonstrated a change in image density after a scan delay of 4 h and 6 h in two consecutive studies [8, 12]. It would be feasible to justify the fact that such a change in density may not be so challenging for clinicians when examining images. Additionally, creation and quality of the image from the trapped electrons that remains on the PSP plates after delayed scanning may be associated with a consequence of 2 key factors [1]: the minimum threshold for the phosphor crystals is required to create a satisfactory diagnostic image, and [2] the effects of specific software and hardware which are automatically able to promote the density to a level that remains diagnostic to the clinician *via* the monitor. The high quality of the images that remain after delayed scan, can be deduced by the special mechanism of PSP image formation. The image formation is efficient with PSP because the phosphor crystal layer absorbs much more radiation than non-screen film; consequently, a lower exposure is required to obtain the same optical density [10, 17-23]. Another point that must be noted is contrast (difference of mean density between steps). In our study, the contrast decreased in all environments after 8 hours reflecting the loss of information and the decrease in contrast that had occurred. Similar results were obtained by Akdeniz *et al*. [7]. Previous studies have reported that one of the principle reasons that result in damaging the latent image in a PSP system is the delay in scanning time, while in the current study, the impact of the variety of ambient light was also evaluated. In accordance with the result of this study, different environments affected the plates similarly. In all environments, the density was reduced over the time. However, in intermediate density (step 2), the trend was different in yellow light environment, and more decrease was detected during the time. This finding shows that this environmental light can adversely impact the PSP plates images with time. It can be because of the different effect of yellow light on trapped electron in these plates. More studies are suggested on this issue. At last, we concluded that PSP images show significant decrease in the density in plates scanned for 10 minutes or longer after exposure which may not be detected clinically. The yellow light environment had the different impact on the quality of PSP images and reduced the contrast of images. While this impact was not detected in other environments, which suggests that the plates cannot be scanned and processed in yellow light environment.

## Figures and Tables

**Fig. (1):**
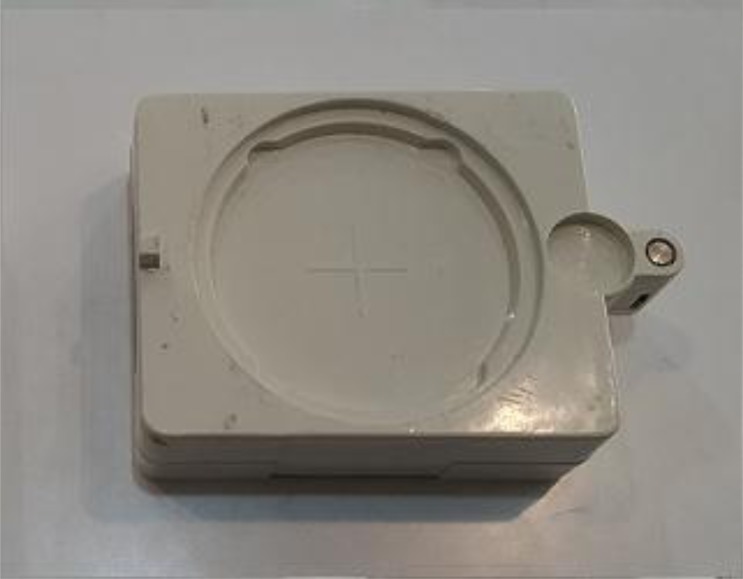
Phantom (for evaluating density and spatial resolution).

**Fig. (2):**
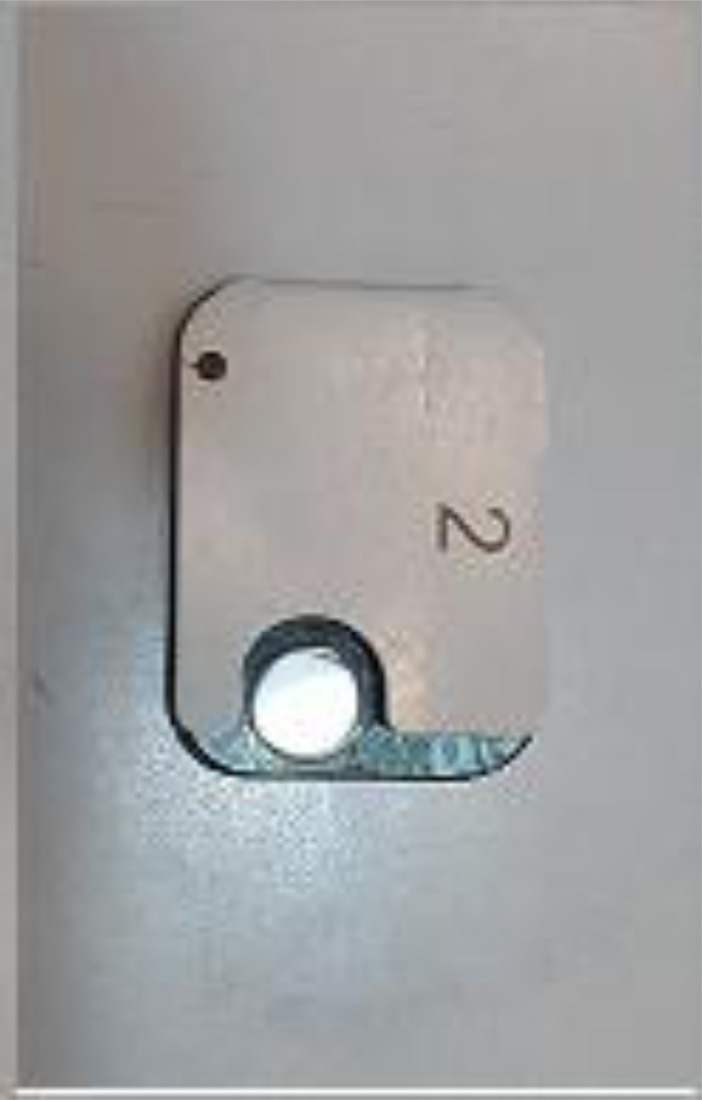
PSP receptor.

**Fig. (5):**
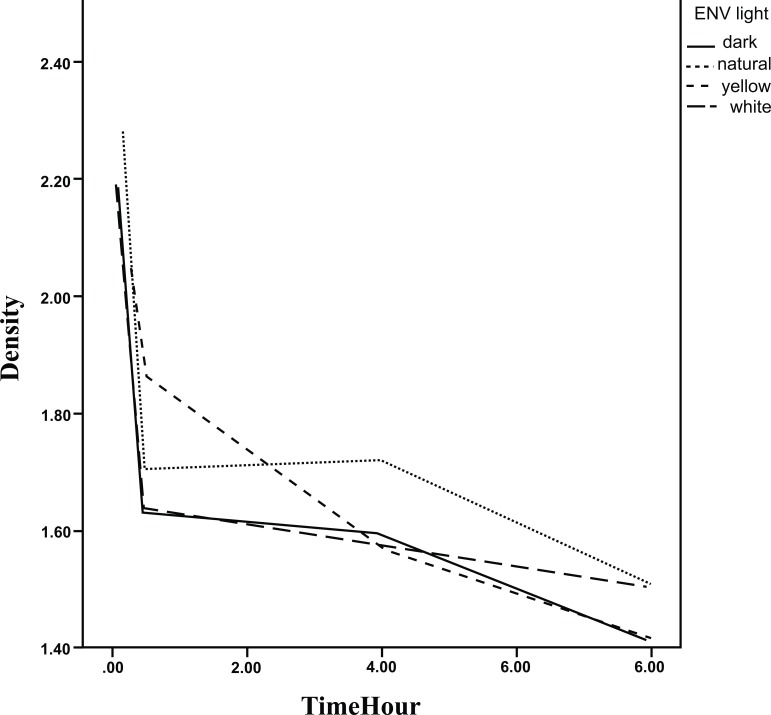
The densities of the plates according to delay in scanning time in different environments.(step 1)

**Fig. (6):**
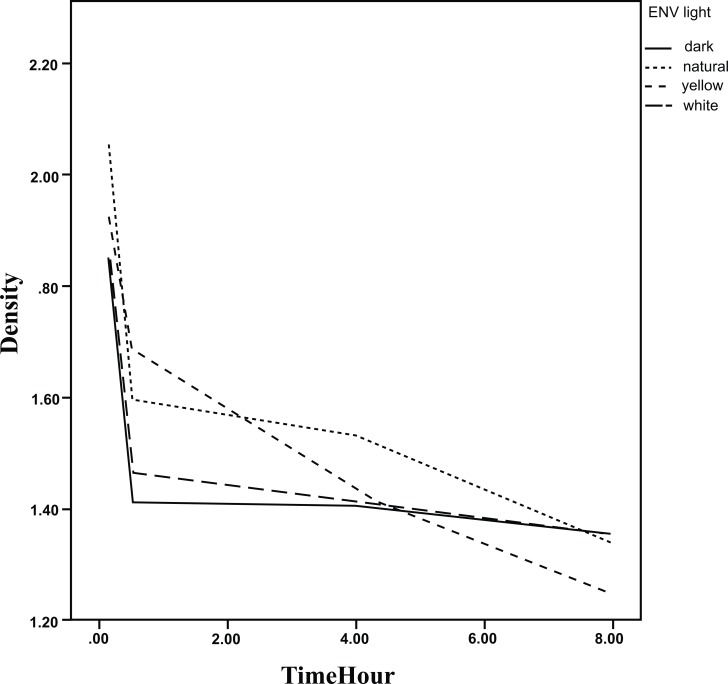
The densities of the plates according to delay in scanning time in different environments.(step 2)

**Fig. (7):**
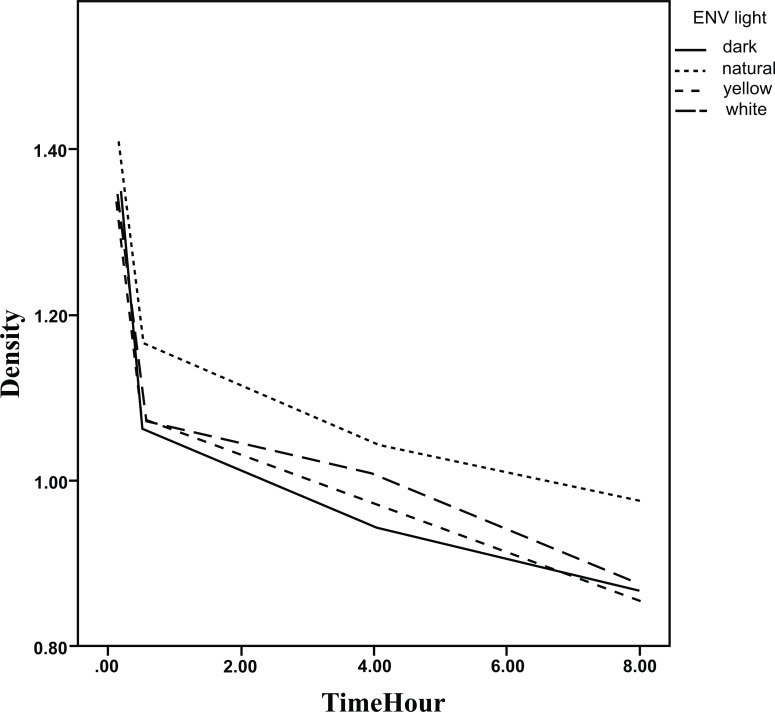
The densities of the plates according to delay in scanning time in different environments.(step 3)

**Fig. (8):**
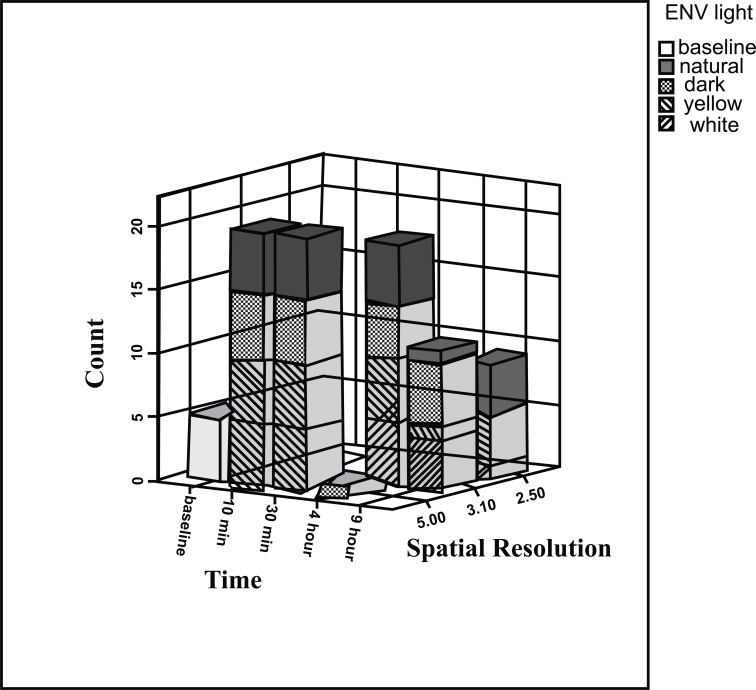
The spatial resolution of the plates according to delay in scanning time in different environments.

**Table 1:** Mean and standard deviation of densities and the paired T-test between each time point and the baseline over the time in different steps.

**Light Env**	**Time**	**Step 1**	***P*-Value**	**Step 2**		**Step 3**	
**Baseline**	**Baseline**	2.272 ± 0.106		1.992 ± 0.153		1.424 ± 0.065	
**Natural**	**10 min**	2.280 ± 0.184	0.938	2.056 ± 0.271	0.705	1.410 ± 0.048	0.699
	**30 min**	1.706 ± 0.266	0.009	1.596 ± 0.276	0.035	1.164 ± 0.071	0.004
	**240 min**	1.718 ± 0.131	0.003	1.530 ± 0.163	0.018	1.042 ± 0.048	<0.001
	**480 min**	1.510 ± 0.140	<0.001	1.336 ± 0.122	0.002	0.974 ± 0.061	<0.001
**Dark room**	**10 min**	2.166 ± 0.056	0.060	1.850 ± 0.043	0.122	1.348 ± 0.029	0.038
	**30 min**	1.630 ± 0.021	<0.001	1.412 ± 0.035	0.001	1.066 ± 0.076	0.003
	**240 min**	1.588 ± 0.204	0.003	1.406 ± 0.218	0.009	0.944 ± 0.091	0.001
	**480 min**	1.408 ± 0.142	0.001	1.354 ± 0.166	0.007	0.868 ± 0.101	<0.001
**Yellow**	**10 min**	2.126 ± 0.093	0.003	1.926 ± 0.140	0.405	1.308 ± 0.070	0.001
	**30 min**	1.860 ± 0.290	0.053	1.688 ± 0.237	0.125	1.076 ± 0.051	<0.001
	**240 min**	1.566 ± 0.092	<0.001	1.428 ± 0.068	0.003	0.978 ± 0.066	<0.001
	**480 min**	1.418 ± 0.116	<0.001	1.248 ± 0.051	0.001	0.854 ± 0.008	<0.001
**White**	**10 min**	2.186 ± 0.059	0.114	1.850 ± 0.080	0.194	1.324 ± 0.028	0.021
	**30 min**	1.638 ± 0.185	0.003	1.464 ± 0.105	0.004	1.074 ± 0.042	0.001
	**240 min**	1.570 ± 0.152	0.001	1.412 ± 0.146	0.008	1.008 ± 0.013	<0.001
	**480 min**	1.502 ± 0.289	0.007	1.350 ± 0.231	0.014	0.876 ± 0.131	0.002

**Table 2:** The results of generalized estimating equations assessing the development of densities based on several light environments over the time.

**Response Variable**	**Parameter**	**Estimate**	**Standard Error**	***P*-Value**
S1	(Intercept)	1.912		
	Light Env.			
	White	-0.002	0.057	0.969
	Yellow	0.081	0.052	0.119
	Natural	0.101	0.071	0.157
	Dark room			
	Time	-0.068	0.011	<0.001
	White* Time	0.009	0.005	0.108
	Yellow* Time	-0.012	0.013	0.401
	Natural* Time	0.001	0.012	0.913
	Dark*Time			
S2	(Intercept)	1.632	0.014	<0.001
	Light Env.			
	White	0.026	0.051	0.618
	Yellow	0.182	0.050	<0.001
	Natural	0.210	0.093	0.024
	Dark room			
	Time	-0.040	0.009	<0.001
	White* Time	-0.004	0.004	0.347
	Yellow* Time	-0.036	0.015	0.018
	Natural* Time	-0.027	0.016	0.086
	Dark*Time			
S3	(Intercept)	1.207	0.014	<0.001
	Light Env.			
	White	0.003	0.019	0.886
	Yellow	-0.007	0.037	0.857
	Natural	0.079	0.031	0.012
	Dark room			
	Time	-0.047	0.005	<0.001
	White* Time	0.004	0.009	0.714
	Yellow* Time	0.001	0.007	0.857
	Natural* Time	0.004	0.004	0.427
	Dark*Time			

**Table 3:** The results of generalized estimating equations assessing the mean densities across three steps over the time.

**Parameter**	**B**	**95% Confidence Interval**		**Std. Error**	
		**Lower**	**Upper**		**Sig.**
**(Intercept)**	1.988	1.951	2.026	.019	<0.001
**Step**					
3	-.700	-.729	-.672	.014	<0.001
2	-.237	-.267	-.207	.015	<0.001
1	Reference category				
**time**	-.001	-.001	-9*10^-4^	1*10^-4^	<0.001
Step 3* time	2*10^-4^	-8*10^-5^	.001	2*10^-4^	.156
Step 2* time	2*10^-4^	1*10^-4^	3*10^-4^	4*10^-5^	<0.001
Step 1* time	Reference category				
